# Supplementary material for: MetaRibo-Seq measures translation in microbiomes
Source: Nat Commun. 2020 Jun 29;11:3268. doi: 10.1038/s41467-020-17081-z (PMC7324362; doi:10.1038/s41467-020-17081-z)
Supplement: Supplementary file 10 — Supplementary Data 7 [file 41467_2020_17081_MOESM10_ESM.zip › File2/Confidence_VeryHigh_Taxonomy/357361_out.krona.html]

Javascript must be enabled to view this page.

members
magnitude
magnitudeUnassigned
count
unassigned
taxon
rank

357361\_out

11


SRS018984\_contig\_number\_contig-80\_13599.158142
1

10
superkingdom
2

10
phylum
1239

10
186801
class

186802
1

SRS075084\_contig\_number\_49856
10
order

2
186803
family

2
genus
572511

SRS018656\_contig\_number\_contig-100\_835.77258SRS019068\_contig\_number\_56553

5
31979
family

5
genus

SRS144560\_contig\_number\_12580
1
1485

4
2292202

SRS012273\_contig\_number\_46072SRS015217\_contig\_number\_contig-100\_380.169101SRS023715\_contig\_number\_contig-100\_601.156963SRS148721\_contig\_number\_48899
species

541000
family
1

1
species
552398

SRS018575\_contig\_number\_contig-100\_173.69755

family
186806
1

genus
1730
1

1
species
1235790

SRS1041032\_contig\_number\_2550
